# Floristic Composition, Diversity, and Regeneration of Woody Plant Species of Dabal Forest in Southeastern Ethiopia: Implication for Conservation

**DOI:** 10.1155/2024/7414375

**Published:** 2024-09-09

**Authors:** Zinab Sherafu, Meseret C. Egigu, Sasikumar J. M.

**Affiliations:** Haramaya University School of Biological Sciences and Biotechnology, P.O. Box 138, Dire Dawa, Ethiopia

## Abstract

Floristic composition, population structure, and regeneration status of woody species of Dabal forest found in East Hararghe zone of Oromia Regional State, Ethiopia, were studied. Vegetation data were obtained from 52 main plots of 20 × 20 m for mature woody species and 5 subplots of 5 × 5 m for seedlings and saplings. Density and dbh of each live woody plant species found in each sample plot were recorded. Frequency, basal area, importance value, and Shannon–Wiener diversity indices were also computed. Totally 59 woody plant species distributed in 33 families were documented. Of these, 45.80, 44.10, and 5.08% were shrubs, trees, and lianas, respectively. Species diversity and evenness indices were 3.56 and 0.87, respectively. Stem count of all woody species was 3379 stems ha^−1^. Out of this, 44.6, 30.8, and 24.59% were seedlings (dbh <3.5 cm), saplings (dbh between 3.5 and 10 cm), and mature (dbh >10 cm) individuals, respectively. Total basal area of all individuals with dbh ≥3.5 cm was 29.88 m^2^ ha^−1^. *Juniperus procera*, *Cupressus lusitanica*, *Eucalyptus globulus*, *Afrocarpus falcatus, Eucalyptus camaldulensis*, *Myrsine africana*, *Maytenus undata*, *Hagenia abyssinica*, *Ekebergia capensis*, and *Cordia africana* were species with top ten IVI. Most of these species were top densest, dominant, and frequent. Analysis of population structure based on pooled densities of all species in each dbh class showed that density of seedling > sapling > mature individuals, signifying healthy population structure with good natural regeneration potential. On individual basis, however, 35.6 and 8.5% of species showed fair and poor regeneration statuses, respectively, that deserve attention for conservation.

## 1. Introduction

Due to its wide altitudinal range and diverse topographic features, Ethiopia is endowed with different ecological conditions that favored the occurrence of various plant life-forms with number of vascular plant species estimated between 6500 and 7000, of which 12% are endemic [1]. Besides wild flora, many crops have genetically diversified in Ethiopia [2]. Based on altitude and climate, vegetation of Ethiopia is categorized into 8 types [3]. Dry evergreen Afromontane forest (occurs between 1800 and 3000 masl) is one of the vegetation types that occupies extensive land mass of the country. Majority of Ethiopian population live and practice cereal-based agriculture in Dry evergreen Afromontane forest agroecology [3]. Ethiopian forests in general and dry evergreen Afromontane forest in particular are under great pressure and highly prone to destruction. In Ethiopia, high rate (_∼_3% per annum) of human population growth [4] coupled with increasing demand for resources resulted in conversion of forest ecosystems to agricultural land and settlement areas [5]. Human-based deforestation of natural forest to fulfill the basic human needs does not take place without cost. Changes in natural ecosystem and loss of biological diversity are the ultimate consequences of deforestation [6, 7].

Forests are important for keeping the wellbeing of humans. To mention few, they play vital roles in terms of maintaining healthy environments through sequestration of carbon dioxide, balancing of atmospheric humidity, upholding soil nutrients, etc. Many people rely on forests for several forest products including food, timber, medicine, etc. [8]. Therefore, it is imperative to know floristic composition, diversity, and natural regeneration status of a given plant community. Knowledge on floristic composition, diversity, and species regeneration potential of a given plant community enables one to formulate relevant conservation strategies. Dry Afromonatane forests are widely distributed in Ethiopia. They are found in northern, northwestern, central, southern, southeastern, and southwestern regions of the country [3]. Although ecological studies have been conducted on these forests in different regions of Ethiopia [9–14], the coverage is far less than expected in southeastern region. Dabal Forest is one of the dry Afromontane forests of the country found in southeastern highlands of Ethiopia. Because dry Afromontane forest ecosystems support large human population, we hypothesized that it is under great pressure from human activities including farming to produce crops and animal husbandry, which will have direct or indirect negative impacts on biodiversity. So far no ecological study has been conducted on this forest. We, as a result, designed this study to investigate the floristic composition, population structure, and regeneration status of woody plant species of this forest so that pertinent management practices are devised for its conservation.

## 2. Materials and Methods

### 2.1. Description of the Study Area

**Figure 1:**
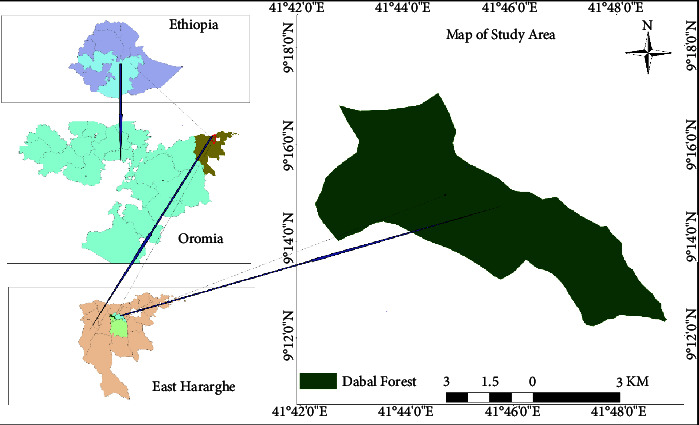
Location map of the study area (Dabal forest).

Geographically, Dabal forest is located between 9°12′00″ and 9°18′00″N latitude, and 41°42′00″ and 41°48′00″E longitude. It is found in East Hararghe zone, Oromia regional state, Ethiopia at about 476 km to the East from Addis Ababa, the capital of Ethiopia (Figure 1). The area covered by this forest encompasses an altitudinal range of 2514 to 3137 masl. Currently, the forest covers about 4088 hectares of landmass, and it is composed of indigenous trees, plantations of fast-growing exotic woody species, and native dense shrubs. The mean annual rainfall of the area ranges from 931 to 1054 mm and has a bimodal distribution [15]. The short rainy season starts in February or March and extends to May, while the long (main) rainy season begins in June and stretches to September, with peak rainfall registered in the months of July and August. The mean annual minimum and maximum temperatures are 10.3 and 18.9°C, respectively [15].

### 2.2. Reconnaissance Survey, Sampling Design, and Vegetation Data Collection

Scouting was made prior to the actual data collection to decide number of line transects to be laid. Five transects each having at least 1 km length were laid in different directions to capture the entire components of the forest. For mature woody species, 52 plots of 400 m^2^ were sampled systematically from the 5 line transects. Each big plot had five 25 m^2^ subplots for saplings and seedlings related data. Mature woody plant species are those with diameter at breast height (dbh) of >10 cm. Saplings had dbh between 3.5 and 10 cm, whereas seedlings were those with dbh <3.5 cm [14, 16]. Mature woody species that were found within the boundaries of the major quadrat, and saplings and seedlings of woody species within the sub-plots were observed and recorded. For mature and saplings, we counted number of stems of each species and measured their stem dbh. Only number of stem of each species was counted for seedlings. The observed plants were temporarily identified on the field by consulting literature including different volumes of Flora of Ethiopia and Eritrea and books published by [17] on useful trees and shrubs of Ethiopia. Plant specimens were then collected and identified in Haramaya University Herbarium. The current botanical names of the collected plants were confirmed by accessing “the plants of the world online” (https://powo.science.kew.org/) website. The identified specimens were finally stored in Haramaya University Herbarium, Ethiopia.

### 2.3. Structural Data Analysis

Vegetation data including density, basal area, and frequency of each species were examined as key structural data by following standard procedures indicated by Diriba et al. [14]. To get density, number of stems of each woody species encountered in all sample plots were counted and expressed as number of stems per hectare. Basal area, which is a cross-sectional area of a stem obtained from a dbh value, was calculated based on the formula for area of a circle with the assumption that the stem cross-section at a height of 1.3 m above ground is circular, and expressed as m^2^ h^−1^. Frequency was measured as the proportion of sample plots in which a given species was observed from the total sample plots. Moreover, relative density, relative frequency, relative dominance, and important value index were computed to analyze structural data. In order to assess species diversity, Shannon–Wiener diversity (*H′*) and Equitability indices were calculated [14].

### 2.4. Analysis of Population Structure and Regeneration Status

Based on the density values of age structure (seedlings, saplings, and mature) of each woody species and combined density values of each age category of all species, the population structures were demonstrated in histograms. Regeneration status of each species and the entire vegetation was then interpreted from the histogram [14, 18]. Regeneration status of a species was considered “good” when number of seedlings is > saplings > mature; fair when seedlings > or ≤ saplings ≤ mature; poor when a species is represented by sapling only (saplings may be ≤ or ≥mature) [14, 19].

## 3. Results

### 3.1. Floristic Composition and Diversity of Woody Species

Results showed that Dabal forest consisted of 59 woody species belonging to 53 genera and 33 families. Life-form distribution of the recorded woody species was 45.8% shrub, 44.1% tree, and 5.08% lianas. Families Fabaceae, Euphorbiaceae, Moraceae, Asteraceae, Rubiaceae, and Rosaceae were the six top species rich families that accounted for 39% of the total recorded species (Table 1). Shannon–Wiener Diversity (*H′*) and Equitability (*E*) indices of this study were 3.56 and 0.87, respectively.

### 3.2. Structural Analysis and Natural Regeneration

Structural analysis including density, frequency, dominance and important value index were computed. Result of this study showed that the density of individual species of all dbh class (i.e., <3.5 cm, ≥3.5 ≤ 10 cm and >10 cm) ranged from 1.0 (for *Ficus vasta*) to 443 (for *Juneprus procera*) stems ha^−1^. Combined density of all woody species having dbh ≥3.5 cm was 1872 stems ha^−1^ and the top ten densest woody plant species are indicated in Table 2.

**Figure 2:**
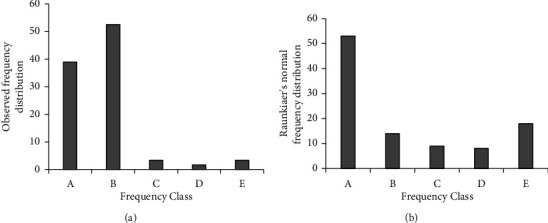
Frequency distributions (a) of the current study and (b) Ranunkiaer's normal frequency.

Frequency of individual species ranged from 4 (for *Ficus vasta*) to 98% (for *Juniperus procera*). The top ten frequent species were *Juniperus procera* (98%), *Cupressus lusitanica* (83%), *Myrsine africana* (63%), *Eucalyptus globulus* (58%), *Maytenus undata* (44%), *Solanum incanum* (33%), *Carissa spinarum* (31%), *Osyris quadripartite* (31%), *Eucalyptus camaldulensis*, and *Lippia abyssinica* (29%) each. Frequency distribution of Dabal forest was categorized into five classes *viz.* A (0–20%), B (21–40%), C (41–60%), D (61–80%), E (81–100%) [20] (Figure 2(a)), and compared with Raunkiaer's normal frequency distribution (Figure 2(b)).

Basal area (dominance) of all individuals having dbh ≥3.5 cm was 29.88 m^2^ ha^−1^. *Juniperus procera*, *Cupressus lusitanica*, *Eucalyptus globulus*, *Afrocarpus falcatus*, *Eucalyptus camaldulensis, Ekebergia capensis*, *Cordia africana*, *Hagenia abyssinica*, *Schefflera abyssinica*, and *Croton macrostachyus* were the ten top dominant species whose basal area accounted for 79.7% of the entire species (Table 3).

Important value index (IVI) was computed from the relative density, relative frequency and relative dominance (relative basal area), and result showed that ten species accounted for more than 50% of the entire species' IVI values combined (Table 4).

**Figure 3:**
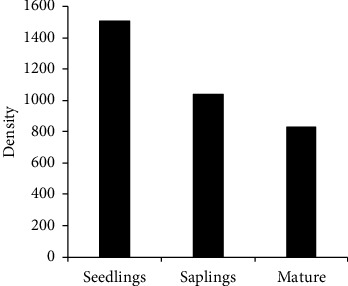
Population structure of Dabal forest with densities of all species combined.

In this study, population structure was depicted using a histogram drawn from density (*Y*-axis) vs three dbh classes (on *X*-axis). Densities of the entire species combined in each dbh class are represented by Figure 3. From this population structure, number of seedlings was largest followed by saplings and mature woody species.

Besides, age structure patterns of individual plant species were assessed and 47.7% of the entire species were found to have population structure similar to Figure 2, whereas 35.6% of the entire species had number of seedlings > or ≤ saplings ≤ mature ones. About 8.5% species were represented by saplings only (Table 5).

## 4. Discussion

### 4.1. Species Composition and Diversity

Knowledge on species composition and diversity of vegetation is important to figure out the stability of an ecosystem. In this study, we recorded 59 wood plant species that belonged to 33 families of which two are gymnosperm. Thirty-nine percent of the recorded species belonged to six families with Fabaceae contributing six species followed by Euphorbiaceae, Moraceae, Asteraceae, Rubiaceae, and Rosaceae each contributing four species. Although different researchers reported different families as the most species rich families, for example, Rosaceae by Sudi et al. [9] and Asteraceae by Yirga et al. [12], several other researchers [11, 13, 14] who conducted research on other dry evergreen Afromontane forests found in different regions of the country reported Fabaceae as the number one species rich family. According to Ricklefs et al. [21], familial species richness may be related to the successful dispersal strategy and ecological amplitude of the family. They further claimed that successful dispersal and wider ecological amplitude are determinants for the existence of a number of growth forms (herbaceous to woody) within a family so that morphologically differentiated groups that fit to varying dispersal and pollination mechanisms are comprised, hence, more number of species within a family. According to Hedberg et al. [22], Fabaceae and Asteraceae are the two species richest families in the Flora of Ethiopia and Eritrea, being represented by 620 and 440 species, respectively. They attributed this familial species richness to their adaptation to successful seed dispersal mechanisms.

Magurran [23] reported that Shannon–Weiner diversity index usually falls between 1.5 and 3.5, and rarely exceeds 4.5. Several similar previous studies conducted in sub-Saharan region [9, 14, 24, 25] also reported Shannon–Weiner diversity index value in the range of 1.5 to 3.5. Thus, diversity index of Dabal forest can be considered as high with each species represented by fair number of individuals. We also analyzed one-sample *T*-test to compare between the species diversity of Dabal forest with similar other studied forests from different corners of the country as representatives, and results showed that species diversity of Dabal forest was significantly (*p* < 0.01) higher than that of Yegof forest (*H*′ = 2.26) studied by Mesfin et al. [1], Kahitassa forest (*H*′ = 2.92) studied by Baymot et al. [26], Gemechis forest (*H*′ = 3.04) studied by Sudi et al. [9], and Wof Washa forest (*H*′ = 3.25) studied by Gebremichael et al. [27]. Thus, Shannon–Weiner index value of this vegetation indicates that Dabal forest is of diverse and heterogeneous plant assemblage [28, 29]. A slight difference between sites in terms of climate, soil nature, and perturbations by biotic factors such as human interference may be the cause for the observed difference in species diversity [12, 18].

### 4.2. Vegetation Structure

With respect to density, *Juniperus procera*, *Cupressus lusitanica*, *Myrsine africana*, *Eucalyptus globulus*, *Maytenus undata*, *Osyris quadripartite*, *Carissa spinarum*, *Eucalyptus camaldulensis*, *Afrocarpus falcatus*, and *Justicia schimperiana* were top densest species that accounted for more than half (52.2%) of the total density of species with dbh ≥3.5 cm. The total density of woody species with dbh ≥3.5 cm recorded in this study appeared to vary from other similar studies. For example, it is higher than what reported by Yineger et al. [30], [898 stems ha^−1^] and by Gebremichael et al. [27] [698 stems ha^−1^] from other similar Afromontane forests of the country. However, it is lower than what reported by Deressa et al. [14] from central highland in Ethiopia. Frequency is suggestive of whether or not a species has a wide occurrence in a community. Based on Raunkiaer's five frequency classes *viz.* A (0–20%), B (21–40%), C (41–60%), D (61–80%), and E (81–100%) [20]; we also analyzed species frequency distribution of Dabal forest and compared it with that of Raunkiaer's normal frequency distribution. Likewise Raunkiaer's frequency distribution, frequency distribution pattern of Dabal forest also had five classes, and in agreement with that of Raunkiaer's normal frequency distribution, suggesting large number (39%) of species in Dabal forest had poor dispersion. According to [14], the difference in density and frequency of a species between regions and between different species in the same region may be due to locational variation, heterogeneity (patchiness) of the environment, and extent of disturbance by abiotic and biotic factors. The sum of basal area of all individual of all species was 29.88 m^2^ ha^−1^. About 80% of the entire species' basal area was contributed just by ten species, namely, *Juniperus procera*, *Hesperocyparis lusitanica*, *Eucalyptus globulus*, *Afrocarpus falcatus*, *Eucalyptus camaldulensis, Ekebergia capensis*, *Cordia africana*, *Hagenia abyssinica*, *Schefflera abyssinica*, and *Croton macrostachyus*. These species are, therefore, the dominant species shaping the environmental conditions in Dabal forest.

Derived from the measures of species density, frequency, and dominance; important value index (IVI) signifies the ecological importance of species in a given ecosystem [18]. Ten species including *Juniperus procera*, *Cupressus lusitanica*, *Eucalyptus globulus*, *Afrocarpus falcatus, Eucalyptus camaldulensis*, *Myrsine africana*, *Maytenus undata*, *Hagenia abyssinica*, *Ekebergia capensis*, and *Cordia africana* were species with top ten values that accounted for 51% of the whole species combined. Most of these species, for example, *Juniperus procera* (98%), *Hesperocyparis lusitanica, Eucalyptus globulus, Eucalyptus camaldulensis, Afrocarpus falcatus, Maytenus undata,* and *Osyris quadripartite* were also within top densest, frequent, and dominant groups. Thus, by virtue of their density, frequency, and basal area; these species are ecologically important to shape the ecosystem under which other companion species are able to survive in the studied area. Ecologically important species can be inferred from their IVI values [18]. The IVI value also indicates how much emphasis should be given to a species for conservation [31]. Overall, the higher IVI, suggests suitable habitat conditions that uphold effective utilization of the available resources, reproduction and growth rate [32].

### 4.3. Species Natural Regeneration

Population structure drawn from the composite density of all species in each dbh class showed that number of seedlings (dbh <3.5 cm) was > saplings (3.5 to 10 cm) > mature (dbh >10 cm) woody species. Regeneration status of a species was considered “good” when number of seedlings is > saplings > mature; “fair” when seedlings > or ≤ saplings ≤ mature; “poor” when a species is represented by sapling only (saplings may be ≤ or ≥ mature) [14, 19]. From the composite population structure, Dabal forest is generally in a good regeneration status. A forest with high number of seedlings followed by saplings and mature individuals is assumed to be healthy and naturally regenerating forest [12, 13, 33–35]. Of course, this may not be taken for granted as there can be mortality difference between size (dbh) classes. Because there can be selective cutting of individuals of certain dbh class for some purposes and also predation by herbivores. Population structure pattern, which is suggestive of good natural regeneration assumes equal mortality among different dbh classes [36]. Population structure patterns of individual species were also analyzed separately and majority (47.7%) of them had good regeneration status while 35.6 and 8.5% of them were in a “fair” and “poor” regeneration statuses, respectively. Two species including *Ficus vasta* and *Gardenia ternifolia* were represented only by mature individuals, suggesting hampered regeneration. Lack of seedlings and saplings for these species shows poor seedling recruitment, which may be due to unavailability of seeds from seed rain and/or soil seed bank. Viability of seeds, favorable environmental conditions, and predation of seeds and/or seedlings may also be the causes for poor seedling establishment [37]. On the other hand, *Argomuellera macrophylla*, *Lippia abyssinica*, *Ocimum gratissimum*, and *Senna didymobotrya* lacked mature individuals, which suggests that they are newly invading species or there is selective cutting of individuals of these species in higher dbh class for some purposes.

## 5. Conclusions

Information on floristic composition, structure, and regeneration status of vegetation reveals dynamics of forest ecosystem for sustainable utilization and conservation. Thus, we studied Dabal forest from this perspective. Results of this study showed that Dabal forest harbored 59 woody plant species distributed in 33 families. This forest was found to be diverse with each species being represented by fair number of individuals. Of the entire species, ten of them accounted for 51% of the IVI value of the whole species combined. Most of these species were also the densest, frequent, and dominant. Results of population structure based on aggregate densities of all species revealed that number of seedlings > sapling > mature ones, implying good regeneration status of this forest ecosystem. However, 35.6 and 8.5% of the species had fair and poor regeneration statuses, respectively. Species with poor regeneration status and low IVI need attention for conservation.

## Figures and Tables

**Table 1:** Species diverse families.

No.	Family	No. of species	Percent	Cumulative percent
1	Fabaceae	6	10.17	10.17
2	Euphorbiaceae	4	6.8	16.97
3	Moraceae	4	6.8	23.77
4	Asteraceae	3	5.08	28.85
5	Rubiaceae	3	5.08	33.93
6	Rosaceae	3	5.08	39.01
7	Cupressaceae	2	3.39	42.4
8	Flacourtiaceae	2	3.39	45.79
9	Oleaceae	2	3.39	49.18
10	Verbenaceae	2	3.39	52.57
11	Myrsinaceae	2	3.39	55.96
12	Celastraceae	2	3.39	55.39
13	Lamiaceae	2	3.39	62.74
14	Myrtaceae	2	3.39	66.13

	Other families (19)	Other species (20)	33.87	100

**Table 2:** Top ten densest woody species with dbh ≥3.5 cm.

Species name	Density (stems ha^−1)^	Rank	Relative percent contribution	Cumulative percentage
*Juniperus procera*	309	1	16.5	16.5
*Cupressus lusitanica*	153	2	8.2	24.7
*Myrsine africana*	96	3	5.1	29.8
*Eucalyptus globulus*	91	4	4.9	34.7
*Maytenus undata*	69	5	3.7	38.4
*Osyris quadripartite*	68	6	3.6	42
*Carissa spinarum*	51	7	2.7	44.7
*Eucalyptus camaldulensis*	50	8	2.6	47.3
*Afrocarpus falcatus*	49	9	2.6	49.9
*Justicia schimperiana*	43	10	2.3	52.2

The rest 49 species				47.8

**Table 3:** Top ten dominant species.

Species name	BA (m^2^ h^−1^)	Rank	%	Cumulative percentage
*Juniperus procera*	9.35	1	31.3	31.3
*Cupressus lusitanica*	3.64	2	12.2	43.5
*Eucalyptus globulus*	2.86	3	9.6	53.1
*Afrocarpus falcatus*	1.7	4	5.7	58.8
*Eucalyptus camaldulensis*	1.4	5	4.7	63.5
*Ekebergia capensis*	1.09	6	3.6	67.1
*Cordia africana*	1.04	7	3.5	70.6
*Hagenia abyssinica*	1.03	8	3.4	74.1
*Schefflera abyssinica*	0.85	9	2.8	76.9
*Croton macrostachyus*	0.83	10	2.8	79.7

The rest 49 species				20.3

**Table 4:** Species with top ten IVI.

Species name	IVI	Rank	%	Cumulative percentage
*Juniperus procera*	55	1	18.3	18.3
*Cupressus lusitanica*	26	2	8.7	27
*Eucalyptus globulus*	19	3	6.3	33.3
*Afrocarpus falcatus*	10	4	3.3	36.6
*Eucalyptus camaldulensis*	9	5	3	39.6
*Myrsine africana*	8	6	2.7	42.3
*Maytenus undata*	7	7	2.3	44.6
*Hagenia abyssinicas*	6.7	8	2.2	46.8
*Ekebergia capensis*	6.4	9	2.1	48.9
*Cordia africana*	6.3	10	2.1	51

The rest 49 species				49

**Table 5:** Plant species with different regeneration statuses.

Regeneration status		
Good	Fair	Poor
*Vachellia abyssinica*	*Faidherbia albida*	*Buddleja polystachya*
*Calpurnia aurea*	*Casimiroa edulis*	*Hagenia abyssinica*
*Carissa spinarum*	*Cordia africana*	*Oncoba spinosa*
*Clutia abyssinica*	*Croton macrostachyus*	*Rhus vulgaris*
*Conyza abyssinica*	*Cupressus lusitanica*	*Tamarix aphylla*
*Dodonaea viscosa*	*Dombeya torrida*	
*Dovyalis abyssinica*	*Ekebergia capensis*	
*Ficus sycomorus*	*Erythrina brucei*	
*Ficus thonningii*	*Eucalyptus camaldulensis*	
*Galiniera saxifraga*	*Eucalyptus globulus*	
*Jasminum floribundum*	*Euclea racemosa*	
*Justicia schimperiana*	*Ficus sur*	
*Maesa lanceolata*	*Juniperus procera*	
*Maytenus arbutifolia*	*Myrsine africana*	
*Maytenus undata*	*Olea europaea*	
*Osyris quadripartita*	*Pittosporum viridiflorum*	
*Phytolacca dodecandra*	*Afrocarpus falcatus*	
*Premna schimperi*	*Rosa abyssinica*	
*Pterolobium stellatum*	*Schefflera abyssinica*	
*Pycnostachys abyssinica*	*Solanecio nandensis*	
*Rhamnus prinoides*	*Syzygium guineense*	
*Ricinus communis*	*Vernonia amygdalina*	
*Rubus steudneri*		
*Rumex nervosus*		
*Rytigna neglecta*		
*Solanum incanum*		

## Data Availability

The data that support the findings of this study are available from the corresponding author upon reasonable request.
